# Digital health-enabled life-course management of endometriosis: from early recognition and assisted diagnosis to symptom monitoring and self-management

**DOI:** 10.3389/fmed.2026.1951037

**Published:** 2026-10-06

**Authors:** QinQin Deng, ZiChen Shao, Min Yang

**Affiliations:** 1 Jiangxi University of Chinese Medicine, Nanchang, Jiangxi, China; 2 Affiliated Hospital of Jiangxi University of Chinese Medicine, Nanchang, Jiangxi, China

**Keywords:** artificial intelligence, digital health, electronic health records, endometriosis, life-course management, mobile health, patient-reported outcomes, telemedicine

## Abstract

Endometriosis is a chronic inflammatory disease that often requires longitudinal care across recognition, diagnostic assessment, symptom control, fertility planning, treatment transitions, and recurrence reassessment. The information required for these decisions is frequently fragmented across patient experience, clinical encounters, imaging, treatment history, and health-system records. This narrative Review synthesizes digital-health applications in endometriosis according to clinical function, data source, and translational maturity. Literature was identified through structured searches of major biomedical databases and targeted reference screening, with emphasis on clinically relevant validation, prospective evaluation, and implementation evidence. The most mature applications include structured electronic symptom capture, electronic patient-reported outcomes, selected app- or telehealth-based support, and digital pain interventions. AI/ML approaches for imaging, risk estimation, molecular classification, fertility outcomes, and recurrence prediction are technically active but are commonly limited by internal validation, uncertain calibration and action thresholds, and sparse prospective clinical-utility evidence. We propose a clinician-supervised life-course framework in which patient-expressed data, clinical and imaging information, treatment history, fertility goals, and follow-up data are integrated to support early recognition, assisted diagnosis, monitoring, shared decisions, and reassessment. The target state is clinical coherence rather than maximal data capture, supported by explicit institutional responsibility for review, escalation, feedback, privacy, equity, and human oversight. Progress toward routine use will depend on external validation, interoperable data architecture, low-burden measurement, prospective pathway evaluation, and accountable implementation.

## 1 Introduction

### 1.1 Endometriosis as a life-course management challenge

Endometriosis is a chronic, estrogen-dependent inflammatory disease characterized by endometrium-like tissue outside the uterus and requires both accurate diagnosis and long-term management across changing symptoms, reproductive goals, treatment responses, and recurrence risk (1). Diagnosis is based on clinical assessment and appropriate imaging, with laparoscopy and histologic assessment used when clinically indicated rather than as an obligatory single diagnostic event (1). Patients may experience cyclic and non-cyclic pelvic pain, gastrointestinal or urinary symptoms, fatigue, subfertility, treatment-related decisions, recurrence concerns, and fluctuating effects on function and quality of life. The ESHRE guideline therefore emphasizes integrated clinical assessment, appropriate imaging expertise, shared decision-making, and attention to symptoms, fertility goals, and long-term care (1). These needs unfold across different stages of life and frequently require information collected outside specialist encounters.

Endometriosis has inflammatory and immune-related features and is influenced by hormonal signalling and genetic susceptibility, while environmental endocrine-disrupting exposures have also been investigated as potential contributors to disease development (2–6). The clinical burden is not determined by lesion extent alone. Symptoms and treatment response may also be shaped by lesion location and phenotype, adhesions, previous surgery, adenomyosis, interstitial cystitis/bladder pain syndrome, pelvic floor dysfunction, gastrointestinal symptoms or coexisting bowel disorders, and psychosocial or lifestyle context. These dimensions are relevant to a life-course digital framework because they affect symptom interpretation, quality of life, fertility decisions, treatment selection, and the meaning of longitudinal change.

Early recognition and continuous management remain difficult because symptoms may be normalized, attributed to other conditions, or recalled incompletely across menstrual cycles (7, 8). First-contact services may lack access to longitudinal symptom patterns, previous treatment responses, or specialist imaging (7, 9, 10). A single biomarker, an isolated mobile application, or an internally validated prediction model cannot address these interconnected clinical and information-continuity challenges alone. The central challenge is to connect patient-generated information, clinical records, imaging, and follow-up data in ways that support timely review, appropriate escalation, and longitudinal reassessment (9–14).

### 1.2 Why digital health is relevant

Digital health is relevant because endometriosis poses recurring clinical challenges in timing, continuity, measurement, and coordination. Risk-based case finding depends on capturing symptoms before specialist review; assisted diagnosis may draw on clinical, imaging, and molecular data; pain management requires repeated assessment; fertility and recurrence management depend on time-stamped follow-up; and self-management requires feedback that is understandable and actionable. A scoping review has already identified digital approaches spanning pain assessment, monitoring, education, and intervention (15). Electronic pain and bleeding diaries and real-time symptom measures demonstrate how episodic recall can be converted into longitudinal data (11–13), while randomized trials of immersive digital therapeutics show that digital tools can also function as active interventions (16, 17).

The evidence remains fragmented by technology and clinical scenario. AI/ML research often focuses on diagnostic or predictive performance; mHealth studies emphasize usability, education, or symptom logging; electronic patient-reported outcome studies focus on measurement; and telemedicine research addresses access and follow-up. Each field answers a partial question. The more clinically relevant question is how these technologies can be combined across the care pathway without overstating diagnostic authority, increasing patient burden, or creating unmanageable data streams (9, 15, 18–20).

### 1.3 Review gap and objective

Several gaps motivate this Review. Many AI/ML studies emphasize discrimination while giving less attention to calibration, external validation, clinical utility, fairness, and workflow integration (21–24). Patient-generated data may improve continuity, but adherence, interpretability, alert thresholds, and response responsibilities are often insufficiently defined (25–27). Evidence for EHR integration, real-world implementation, privacy, equity, and regulation is less developed than technical model building (14, 24, 28–31). In addition, existing reviews commonly organize the field by technology rather than by clinical function (15, 18, 19).

The objective of this Review is to synthesize digital health in endometriosis through a life-course management lens. It examines how digital tools may support early recognition, risk-based case finding, assisted diagnosis, symptom monitoring, pain management, treatment follow-up, fertility counselling, recurrence reassessment, and self-management. It also distinguishes promising research applications from tools that are closer to implementation and identifies the validation, governance, and clinical conditions required for responsible translation.

## 2 Approach to the narrative synthesis

### 2.1 Review design

This article is a narrative Review with a structured, clinically oriented synthesis. It was designed to integrate a heterogeneous literature spanning diagnostic technologies, patient-generated data, digital interventions, longitudinal records, and implementation rather than to estimate a pooled treatment or diagnostic effect. The review therefore used a transparent structured-search and eligibility-screening process while retaining an interpretive narrative synthesis rather than a systematic-review design.

### 2.2 Search strategy

Exported structured searches were conducted in PubMed/MEDLINE and Web of Science Core Collection from database inception through 22 July 2026; the final exported searches were conducted on 22 July 2026. The core search combined “endometriosis” with digital-health concepts including “digital health,” “artificial intelligence,” “machine learning,” “mobile health,” “electronic patient-reported outcome,” “telemedicine,” “digital therapeutics,” “electronic health record,” “real-world data,” “wearable,” and “digital decision support.” To improve sensitivity for the recognition and diagnostic pathway, targeted supplementary concepts included “diagnostic imaging,” “ultrasonography,” “magnetic resonance imaging,” “early detection,” “screening,” “diagnostic delay,” “symptom checker,” and “risk triage.” Reference lists of relevant clinical guidelines, systematic or scoping reviews, and key primary studies were screened for additional publications. Database-specific Boolean search strategies and search records are provided in Supplementary Table S1.

### 2.3 Eligibility criteria

#### 2.3.1 Inclusion criteria

Sources were eligible when they (1) were directly relevant to endometriosis or to a methodological or implementation issue directly applicable to the endometriosis care pathway; (2) addressed at least one prespecified clinical function, including early recognition or triage, assisted diagnosis, longitudinal symptom measurement, digital intervention delivery, treatment or fertility follow-up, recurrence reassessment, self-management, or system-level integration; and (3) comprised clinical guidelines, systematic or scoping reviews, validation studies, randomized or prospective evaluations, cohort studies, implementation research, or other peer-reviewed evidence directly relevant to the review objectives. Broader digital-health, AI, interoperability, governance, or implementation literature was eligible only when it directly informed a methodological or translational issue relevant to endometriosis care.

#### 2.3.2 Exclusion criteria

Sources were excluded from the core synthesis if they (1) were duplicate records; (2) lacked direct relevance to endometriosis or to a prespecified methodological, clinical, or implementation function; (3) consisted of purely technical model development without a clinically interpretable use case; or (4) did not provide sufficient information to support interpretation within the clinical life-course framework.

### 2.4 Study selection and screening

Database searches and manually consolidated search records identified 400 records, with 7 additional benchmark review or reference-tracing sources identified through supplementary screening. After removal of 0 duplicate records, 407 source records were screened against the predefined eligibility criteria. Of these, 391 sources were retained in the core candidate library and underwent PDF-based full-text assessment, and 67 sources were cited in the final core narrative synthesis.

Title/abstract screening and initial classification were performed by QD with methodological support from ZS. Potentially eligible full texts and the final source set were reviewed by QD and ZS, and uncertain eligibility or interpretation decisions were discussed with MY until consensus was reached.

Because this was a narrative Review, study selection was purposive rather than exhaustive, and duplicate independent screening was not undertaken. Formal risk-of-bias assessment, quantitative pooling, and protocol registration were also not performed.

### 2.5 Evidence synthesis framework

Evidence was synthesized along three prespecified axes: clinical function, data source, and translational maturity. Clinical functions comprised recognition and triage, assisted diagnosis, longitudinal symptom monitoring, intervention delivery, treatment and fertility follow-up, recurrence reassessment, self-management, and health-system integration. Data sources were categorized as patient-expressed or patient-generated, clinical, imaging, molecular, and system-level data. Translational maturity was classified according to the furthest stage demonstrated by the available evidence: feasibility/model development, internal validation, external validation, prospective clinical evaluation, or routine deployment with monitoring. Conclusions were qualitative and were weighted by evidence directness, validation stage, prospective evaluation, and relevance to an actionable clinical pathway rather than by headline performance metrics alone.

## 3 Digital health technology spectrum and data architecture

### 3.1 Scope and terminology

In this Review, digital health refers to tools, systems, and data practices that capture, integrate, analyze, communicate, or act on information relevant to endometriosis care. Patient-facing technologies include mobile health (mHealth) applications, symptom diaries, electronic patient-reported outcomes (ePROs), digital education, decision aids, and digital therapeutics. Clinician-facing technologies include AI-assisted imaging, clinical risk models, dashboards, and remote follow-up workflows. Infrastructure-level technologies include electronic health records (EHRs), administrative data, real-world data (RWD), registries, interoperability standards, and governance processes.

Population screening, risk-based case finding, risk triage, and diagnostic support should not be treated as interchangeable. In this Review, early recognition refers to identifying patterns that justify clinical attention; case finding refers to targeted identification among symptomatic or higher-risk individuals; risk triage refers to prioritization for clinician review, imaging, or referral; and assisted diagnosis refers to tools that support, but do not replace, clinical assessment. General online information, social media content, and chatbot outputs are included only when they influence education, symptom interpretation, self-care, or clinical communication. Studies of online videos and generative AI responses illustrate the need to evaluate information quality, reliability, and safety (32, 33).

### 3.2 Data sources and connected architecture

Patient-generated data include pain intensity and location, bleeding patterns, cycle timing, medication use, activity limitations, flare triggers, treatment acceptability, sexual function, fertility intentions, and patient priorities. These data should preserve direct patient expressions wherever possible rather than relying exclusively on clinician-coded summaries. In the language of Clinical Phenomenological Data, clinically relevant experience includes symptoms, function, uncertainty, treatment burden, contextual influences, and the patient's own interpretation of how illness affects daily life (34). ePROs and electronic diaries convert episodic recall into time-stamped trajectories and can make within-person change visible (11–13). Clinical data include history, examination findings, prior surgery, medication exposure, fertility history, comorbidities, treatment response, and specialist assessment. A coherent longitudinal record should retain the relationship between these patient-expressed experiences and the clinician's subsequent interpretation and action.

Where clinically justified, the longitudinal profile may also include family history or genetic-risk information, relevant environmental-exposure history, and patient-generated lifestyle variables such as diet and nutrition, physical activity, sleep quality, stress, and mental well-being. These variables should be treated as contextual or hypothesis-relevant data rather than assumed causal determinants, and their collection should be proportionate to the clinical decision being supported. In particular, digital systems should avoid expanding data capture simply because a variable is technically measurable; clinical relevance, patient burden, consent, and data minimization remain necessary criteria (28, 29).

These sources are complementary rather than interchangeable: patient-generated data describe lived experience, imaging supports anatomical characterization, molecular or genetic data may identify biological or susceptibility signals, and EHR/RWD describe care pathways at scale. Contextual lifestyle and exposure data may refine interpretation in selected use cases but require particularly careful attention to causal uncertainty, privacy, and patient burden.

### 3.3 Technology classes and clinical functions

AI/ML occupies a prominent position in the literature, particularly for imaging, molecular classification, clinical risk prediction, and outcome modelling. Systematic reviews show substantial technical activity but also underline that performance must be interpreted according to data source, validation stage, and clinical context (18, 35, 36). mHealth contributes through accessibility, education, symptom capture, behavioral support, and continuity, although app quality and evidence of benefit remain variable (19, 37, 38). ePROs provide a measurement and communication layer, while digital therapeutics and telehealth interventions add active treatment or support components (20, 39). EHR/RWD support phenotype validation and longitudinal system-level analysis, but their usefulness depends on validated definitions and data quality (9, 10).

The same technology can serve different clinical functions. A mobile application may provide education, collect symptoms, support treatment adherence, or deliver an intervention. An AI model may classify images, estimate risk, predict recurrence, or identify likely treatment outcomes. Clinical value therefore depends less on the technology label than on the decision it is intended to support, the population in which it is used, and the response pathway connected to its output (14, 23, 24). The overall architecture is shown in Figure 1, while Table 1 maps the major digital-health modalities to their clinical functions across the life course.

**Figure 1:**
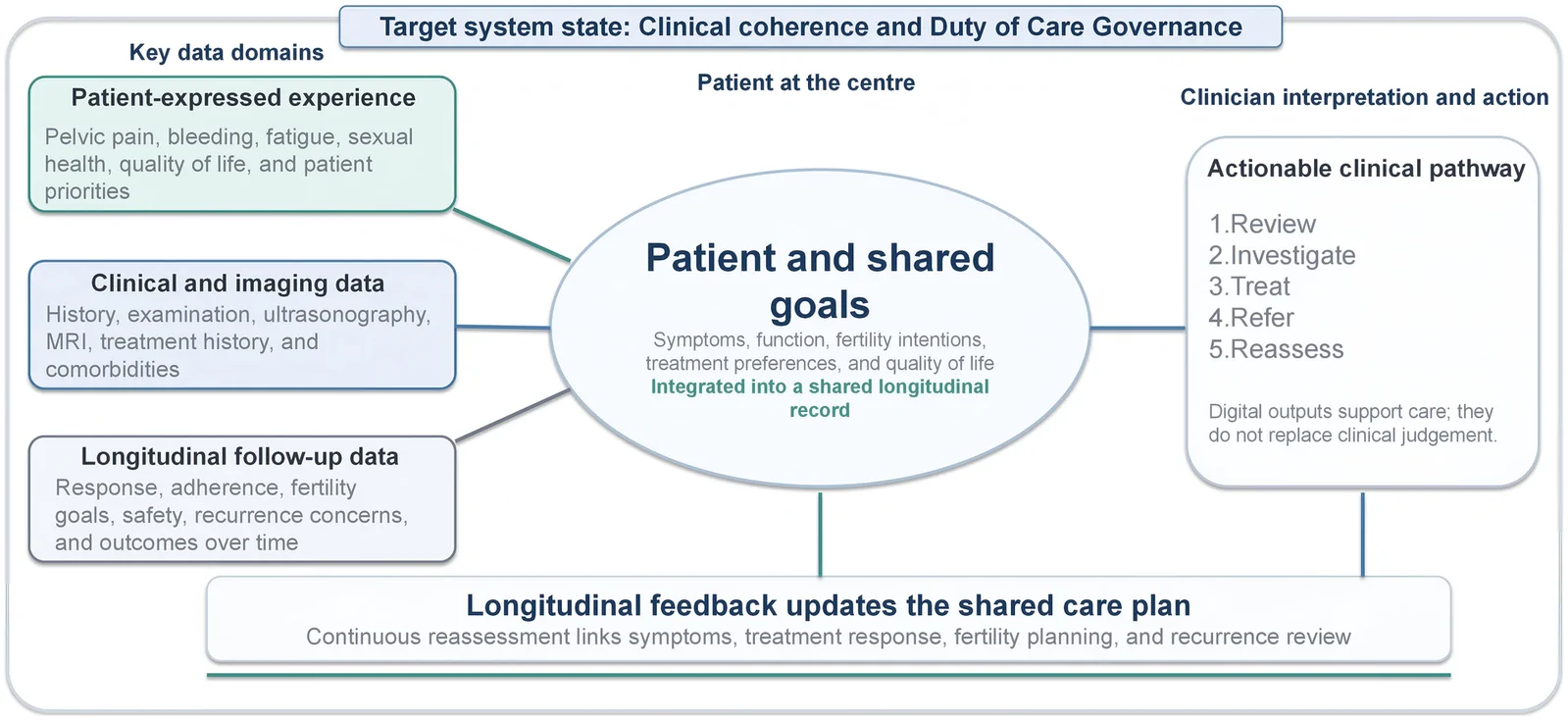
Target state for digital health-enabled life-course management of endometriosis. Patient-expressed experience, clinical and imaging data, and longitudinal follow-up information are integrated around patient goals within a shared longitudinal record. Clinician interpretation links these data to review, investigation, treatment, referral, and reassessment, while longitudinal feedback continuously updates the shared care plan. Clinical coherence represents the target system state, and Duty of Care Governance defines institutional responsibility for data review, escalation, feedback, continuity, and accountable clinical action.

**Table 1:** Digital-health technologies across the endometriosis life course.

Technology	Early recognition	Assisted diagnosis/risk	Monitoring and intervention	Follow-up, fertility, and recurrence	Representative evidence
AI/ML	Risk estimation and prioritization	Imaging, clinical, and molecular models	Dynamic risk update and phenotyping	Outcome and recurrence prediction	(18, 21–24, 35, 36, 45)
mHealth	Symptom capture and education	Referral preparation and decision support	Diaries, self-management, targeted interventions	Medication, visit, and goal tracking	(19, 28, 37, 38, 51, 53)
PRO/ePRO	Symptom burden and patient priorities	Baseline characterization	Pain, function, treatment response, and quality of life	Longitudinal outcomes and tele-follow-up	(11–13, 20, 25–27, 39
EHR/RWD	Population signals and case finding	Clinical phenotyping	Care-pathway and safety analysis	Longitudinal outcomes and learning health systems	(9, 10, 14, 31, 64)
Telemedicine and digital therapeutics	Access and early contact	Care coordination	Remote intervention and supervised support	Remote follow-up and escalation	(16, 17, 20, 26–28, 39, 54–57)

AI, artificial intelligence; EHR, electronic health record; ePRO, electronic patient-reported outcome; ML, machine learning; PRO, patient-reported outcome; RWD, real-world data.

### 3.4 Evidence maturity and translational readiness

Technical function is not equivalent to clinical function, and internal validation is not equivalent to clinical readiness. Early-stage studies may demonstrate feasibility, usability, feature extraction, or model development. Intermediate evidence includes psychometric testing, internal validation, or pilot clinical evaluation. More mature evidence requires external validation, prospective evaluation, clinical utility, safety assessment, patient and clinician acceptability, equity analysis, workflow testing, and post-deployment monitoring (21–24, 30).

This maturity gradient should determine the strength of claims. AI/ML studies should be judged by calibration, transportability, utility, and fairness in addition to discrimination. ePRO studies should be judged by validity, burden, interpretability, and defined response pathways. mHealth interventions should be judged by engagement, clinical benefit, privacy, and integration with care. EHR/RWD studies require validated phenotypes, transparent missing-data handling, representativeness, and governance. The purpose of this framework is to distinguish useful research signals from implementation-ready tools (14, 21, 22, 24–28, 30, 31).

## 4 Digitally enabled early recognition and risk-based case finding

### 4.1 From delayed recognition to structured information

Endometriosis is difficult to recognize early because symptoms are heterogeneous, fluctuate across cycles, overlap with gynecological and non-gynecological conditions, and may be normalized by patients or clinicians (1, 7, 8). Digital tools cannot establish the diagnosis in isolation, but they can reduce information loss before specialist assessment. Cycle-aware symptom capture, structured questionnaires, and longitudinal pain trajectories can help organize the clinical history and identify patterns that warrant review (8, 11–13, 40).

Primary care is a critical setting for this early-recognition function because repeated presentations of pelvic pain or menstrual symptoms may precede specialist referral. By analogy with recent work using Primary Ovarian Insufficiency as a sentinel condition for interpreting menstrual abnormalities in primary care (41), endometriosis can be treated as a sentinel diagnostic consideration when persistent or recurrent pelvic pain, dysmenorrhea, dyspareunia, infertility concerns, or cyclic gastrointestinal or urinary symptoms are documented. A digital system can make these longitudinal patterns visible and support predefined decisions to reassess, investigate, or refer, while leaving diagnostic interpretation with the clinician.

The central design requirement is safety-netted triage. A digital output should indicate what action follows, who reviews the information, which red flags require urgent assessment, and when uncertain or lower-probability presentations should be reassessed. This framing reduces the risk that a negative digital score leads to false reassurance or that a low-specificity tool causes indiscriminate referral. Figure 2 summarizes this safety-netted pathway from symptom capture and risk stratification to clinician review, specialist assessment, and reassessment (1, 8, 24, 29, 40).

**Figure 2:**
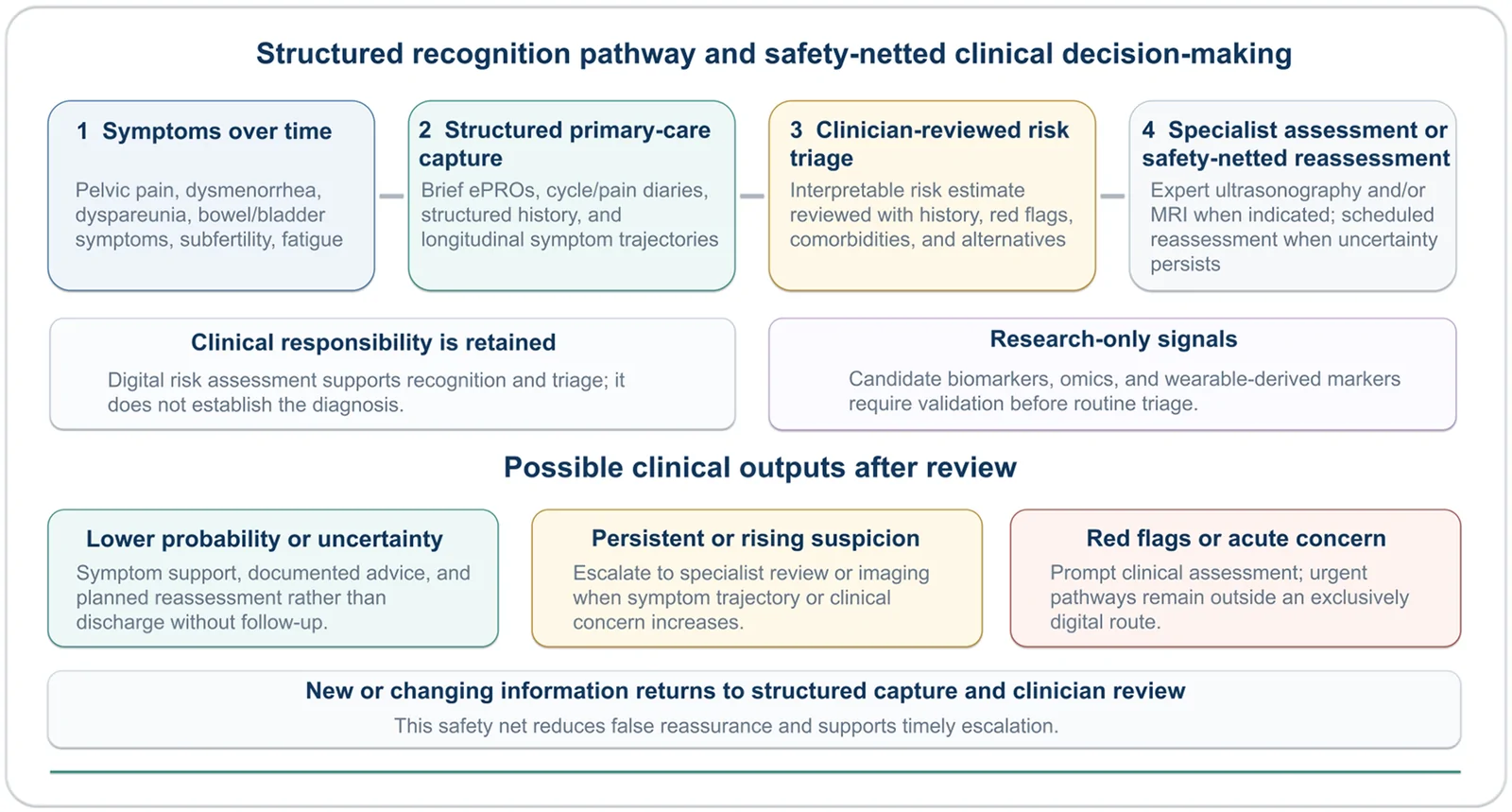
Structured pathway for digitally enabled early recognition, clinician-reviewed triage, and safety-netted reassessment in endometriosis. Longitudinal symptoms are captured in primary care through structured patient-reported information and reviewed within a clinician-supervised risk-triage process. Patients with persistent suspicion may proceed to specialist assessment and clinically indicated imaging, whereas uncertain presentations undergo planned reassessment. Candidate biomarkers, omics data, and wearable-derived signals remain research-stage inputs. Digital risk assessment supports recognition and triage but does not establish the diagnosis, and red flags or acute concerns require prompt clinical assessment outside an exclusively digital pathway.

### 4.2 Symptom capture and patient-facing triage

Symptom checkers and structured screening measures may help patients decide when to seek care or help clinicians prioritize referral, but their role should remain explicitly supportive. A clinical-vignette study illustrates the potential of digital symptom checking in reproductive health while also showing why outputs require clinical interpretation (40). A scoping review of endometriosis screening measures similarly suggests that structured tools may support earlier recognition, but their value must be tested within realistic pathways rather than as standalone tests (8).

### 4.3 Biomarkers, wearables, and computational risk stratification

Research is expanding toward combinations of symptoms, demographics, biomarkers, and digital measurements. Studies of urinary microRNAs, inflammatory biomarkers, and a wearable in-pad platform illustrate potential routes toward less invasive detection (42–44). These approaches remain at different stages of analytical and clinical validation. Before any population-level use, candidate tools require reproducible measurement, clinically relevant thresholds, external validation, assessment of false-positive and false-negative consequences, and a clearly defined route to further assessment.

The strongest near-term role for digital early recognition is therefore structured symptom capture and clinician-reviewed risk triage. Biomarker-enhanced and wearable approaches are promising research directions, but their diagnostic authority should not be inferred from feasibility or internal model performance (7, 8, 22, 24, 40).

## 5 Artificial intelligence and machine learning for assisted diagnosis and prediction

### 5.1 Evidence landscape and validation hierarchy

AI/ML studies in endometriosis use clinical variables, imaging, molecular and omics signatures, and patient-generated data. Potential applications include recognition and triage, imaging support, phenotyping, treatment-response prediction, fertility-related prediction, and recurrence estimation. The heterogeneity of input data and clinical targets makes direct comparison difficult. Model appraisal must therefore begin with intended use, source population, reference standard, and validation design rather than headline accuracy alone (21, 22, 24). Figure 3 summarizes the principal data modalities, clinical targets, and validation hierarchy for AI/ML applications.

**Figure 3:**
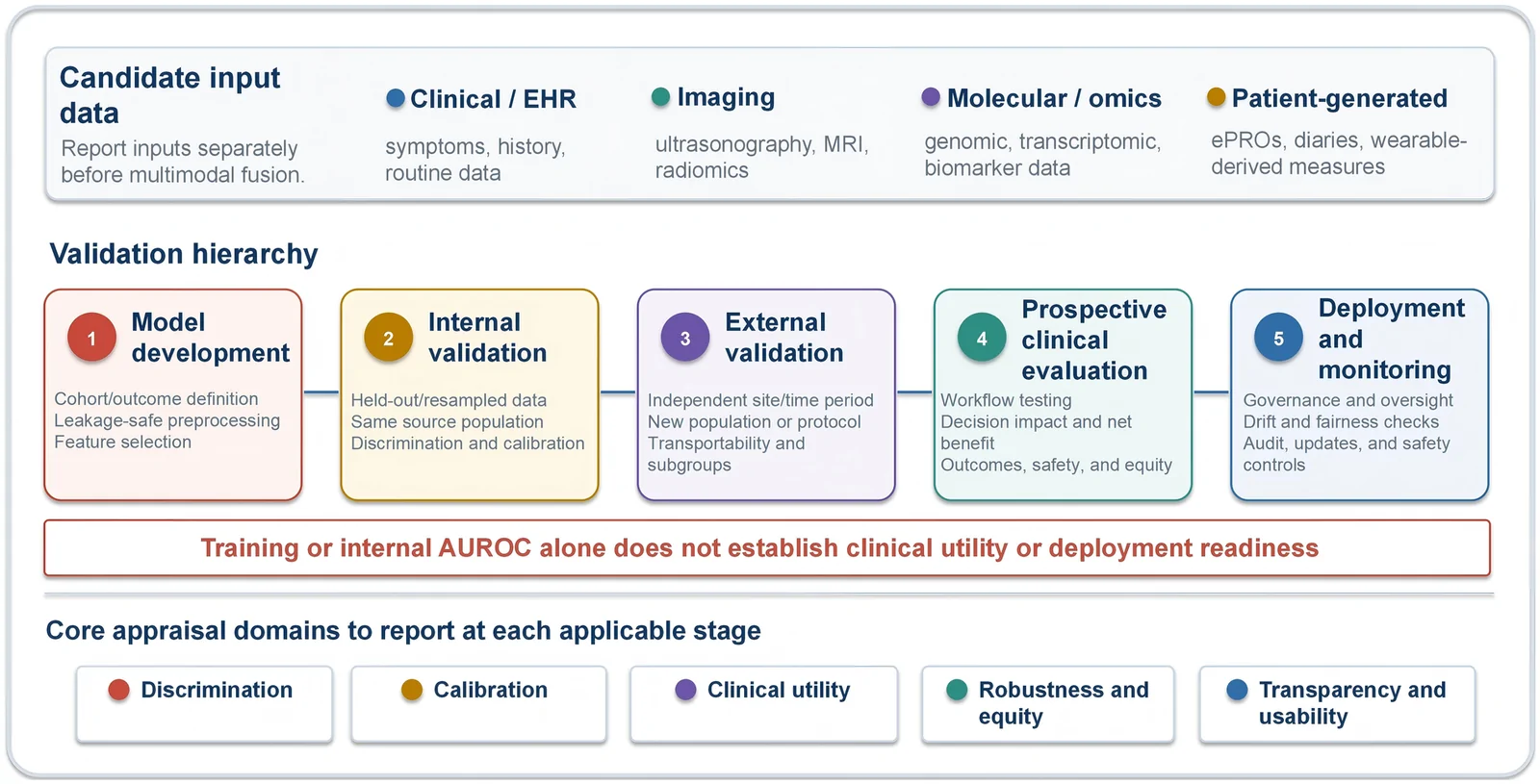
Validation hierarchy and appraisal framework for artificial intelligence and machine-learning applications in endometriosis. Candidate inputs may include clinical and electronic health record data, imaging, molecular or omics data, and patient-generated information. Evidence maturity progresses from model development and internal validation to independent external validation, prospective clinical evaluation, and deployment with ongoing monitoring. Clinical appraisal should extend beyond discrimination to calibration, clinical utility, robustness and equity, transparency, and usability. Training performance or internally validated AUROC alone does not establish clinical utility, transportability, or deployment readiness.

### 5.2 Imaging AI and assisted diagnosis

Imaging-based AI has been explored for ultrasonography and MRI. Systematic reviews and comparative analyses show substantial methodological activity, including models for rectosigmoid deep endometriosis, ovarian endometriosis, and deep infiltrating disease (18, 35, 45–47). The clinical meaning of model performance depends on patient spectrum, imaging protocol, operator expertise, the reference standard, and whether evaluation occurred in the same setting as development. Human-reader comparison should also distinguish assistance from replacement: an algorithm that improves consistency or prioritizes difficult cases may be clinically useful even if it does not outperform expert imaging in every context (21–24).

### 5.3 Clinical, molecular, and multimodal models

Beyond imaging, models have combined symptoms, history, laboratory variables, biomarkers, transcriptomic signals, and other omics features. Systematic and narrative reviews describe high apparent performance in some datasets, particularly in imaging or molecular discovery settings, but also substantial heterogeneity and risk of overfitting (18, 48). Clinical-variable models may be more deployable when they use routinely available inputs, but they still require calibration and transportability testing. Molecular and omics models should generally be treated as hypothesis-generating until biological reproducibility, pre-analytical stability, and prospective clinical validity are established (21, 22, 24).

AI has also been applied to quality-of-life determinants and proposed for biomarker-enhanced screening (49, 50). These applications broaden the field beyond binary diagnosis, but they increase the need to define the decision target. Predicting symptom burden, quality of life, disease phenotype, or referral need are distinct tasks and should not be presented as interchangeable evidence of diagnostic performance.

### 5.4 From model performance to clinical readiness

Internal resampling, cross-validation, and random train-test splits from the same source population do not establish transportability (21, 22). Independent external validation is necessary but still insufficient if calibration, clinical utility, interpretability, subgroup performance, workflow effects, and post-deployment monitoring are not addressed (21–24, 30). A clinical-readiness review highlights this gap between algorithm development and usable endometriosis tools (36). Prospective evaluation should determine whether an AI-supported pathway changes referral quality, diagnostic delay, patient outcomes, workload, or inequity rather than merely reproducing retrospective labels (23, 24). Table 2 provides a structured framework for appraising the main AI/ML evidence groups and their translational limitations.

**Table 2:** Appraisal framework for AI/ML evidence in endometriosis.

Evidence group	Typical inputs	Clinical purpose	Key appraisal questions	Representative evidence
Imaging AI	Ultrasound or MRI images and extracted features	Assisted detection and phenotyping	External validation; reader comparison; workflow integration; spectrum effects	(21, 22, 24, 35, 45–47)
Clinical-variable models	Symptoms, history, demographics, routine laboratory data	Risk estimation, triage, and outcome prediction	Calibration; transportability; missingness; clinically useful thresholds	(8, 21, 22, 40, 43, 49)
Molecular or omics ML	Genomic, transcriptomic, proteomic, metabolomic, or biomarker data	Non-invasive diagnosis and biological stratification	Batch effects; biological reproducibility; pre-analytical stability; prospective validation	(18, 22, 24, 42, 48, 50)
Multimodal models	Combined clinical, imaging, molecular, and patient-generated data	Integrated diagnosis, phenotyping, or longitudinal prediction	Incremental value; data availability; interoperability; explainability; fairness	(18, 21–24, 35, 36)

AI, artificial intelligence; ML, machine learning; MRI, magnetic resonance imaging.

## 6 Electronic patient-reported outcomes, symptom monitoring, and digital pain management

### 6.1 Why patient-reported data are central

Pain, function, fatigue, bleeding, treatment experience, sexual health, and patient priorities are central to endometriosis care but are often incompletely captured during episodic visits. ePROs and digital diaries can make symptom trajectories visible over time. Their clinical value depends on four conditions: the measure is valid; data entry is sufficiently brief; changes are interpretable; and an agreed response pathway connects monitoring to patient feedback or clinical review (25–27).

### 6.2 Measurement tools and longitudinal trajectories

Electronic pain and bleeding diaries and real-time symptom measures provide a measurement foundation for longitudinal assessment (11–13). The 17-item electronic Endometriosis Pain and Bleeding Diary (EPBD) was developed using clinician input, five patient focus groups (*n* = 38), and iterative cognitive interviews (*n* = 22), followed by psychometric evaluation in a multicentre usual-practice study (11). Item-total correlations for pain items ranged from 0.40 to 0.89, correlations with the modified Brief Pain Inventory-Short Form pain-intensity score ranged from 0.46 to 0.61, and test–retest intraclass correlation coefficients for numeric pain ratings were approximately 0.59–0.72, supporting construct validity and generally acceptable reliability while leaving responsiveness and optimal scoring incompletely established (11).

An endometriosis-specific experience-sampling method (ESM) ePRO was subsequently developed through qualitative work and evaluated prospectively using smartphone prompts delivered 10 times daily for one week (12, 13). In the psychometric study, 28 participants completed the protocol, overall prompt compliance was approximately 52%, concurrent validity was strong against several symptom and quality-of-life measures, and internal consistency was reported as good for abdominal symptoms, general somatic symptoms, and positive affect and excellent for negative affect (12). These results support real-time symptom characterization but also demonstrate the potential burden of high-frequency sampling. Such instruments can support baseline characterization, within-person change detection, and treatment review, but their clinical use requires predefined interpretation and response rules. Tele-PROM experience further suggests that remote patient-reported information can support outpatient follow-up when clinicians understand how to interpret and act on it (20). Recording frequency should therefore be matched to the decision being supported rather than defaulting to continuous monitoring (25–27).

### 6.3 Digital pain interventions and supervised support

Digital pain management extends beyond data collection. Reviews describe approaches that combine assessment, education, monitoring, and intervention (15). Randomized evaluations of immersive digital therapeutics and telehealth cognitive behavioural therapy suggest that structured digital interventions may improve pain-related outcomes or quality of life in selected patients (16, 17, 39). However, digital pain scores should not be interpreted as a direct surrogate for lesion activity. Pain severity and treatment response may be influenced by lesion location and phenotype, adhesions, the effectiveness and sequelae of previous surgery, adenomyosis, interstitial cystitis/bladder pain syndrome, pelvic floor dysfunction, gastrointestinal symptoms, and psychosocial context. A clinically useful digital pain pathway should therefore capture relevant comorbidity and treatment-history context and should trigger clinical reassessment when a symptom trajectory is discordant with the expected course. These findings support digital delivery as a component of multimodal care, but durability, patient selection, adherence, and integration with medical or surgical treatment require further study (26, 27).

### 6.4 Closing the monitoring loop

A clinically useful monitoring system is a loop rather than a one-way data repository. Patient-generated information is captured, summarized longitudinally, interpreted using within-person change and prespecified thresholds, and returned through patient-facing and clinician-facing channels. The resulting action may include reassurance, self-management support, treatment review, remote contact, referral, or urgent assessment. Red flags and acute deterioration must remain outside an exclusively digital pathway. Figure 4 illustrates this closed-loop pathway, and Tables 3, 4 distinguishes validated patient-facing measurement instruments from digitally delivered interventions and remote-care platforms (25–27).

**Figure 4:**
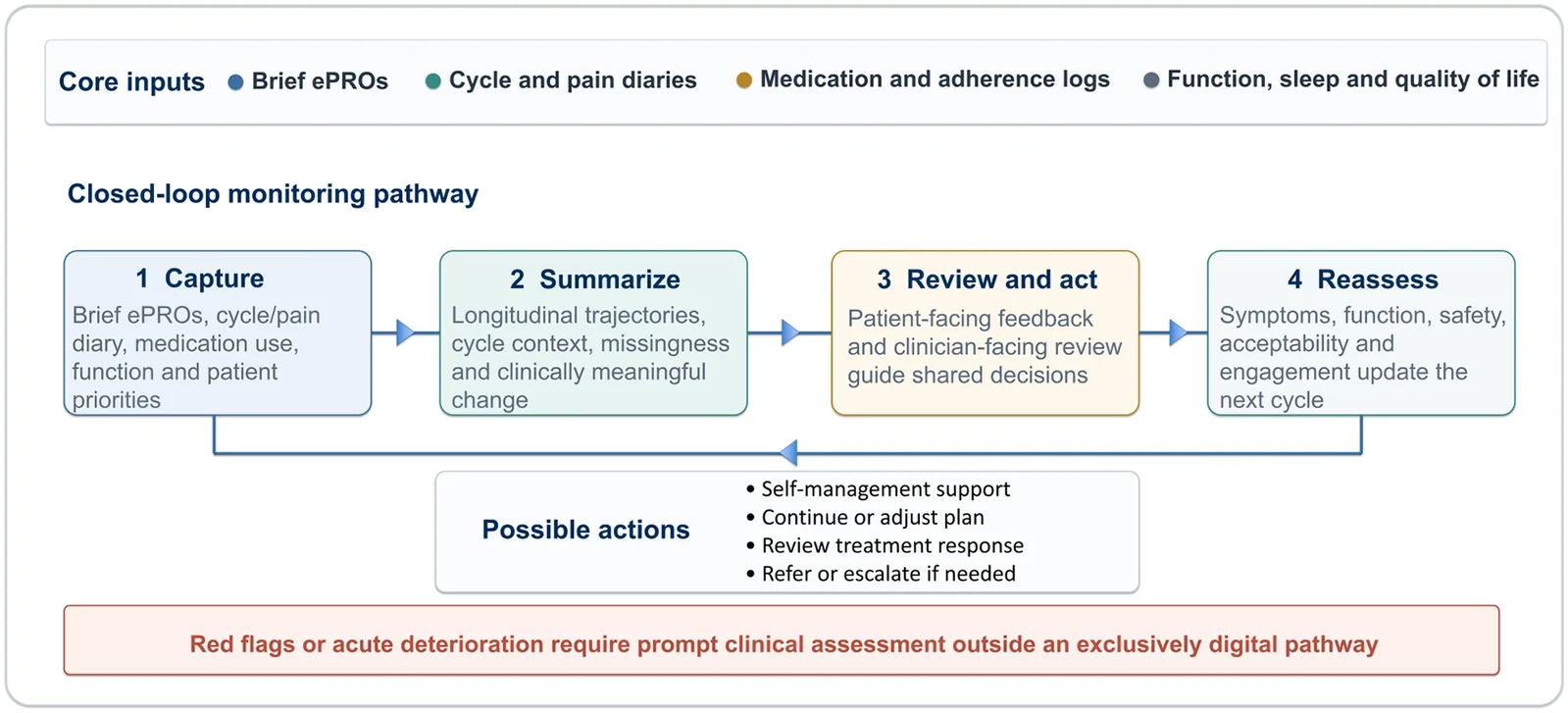
Closed-loop ePRO-enabled symptom monitoring and digital pain-management pathway in endometriosis. Brief ePROs, cycle and pain diaries, medication and adherence logs, and measures of function, sleep, and quality of life are captured and summarized into longitudinal trajectories. Clinician-facing review and patient-facing feedback support shared decisions and individualized actions, followed by reassessment of symptoms, function, safety, acceptability, and engagement. Updated outcomes inform subsequent monitoring cycles. Red flags or acute deterioration require prompt clinical assessment outside an exclusively digital pathway.

**Table 3:** Validated patient-reported measurement instruments.

Original instrument title	Development/validation source	Primary clinical use	Reliability/validity evidence	Main limitation
Electronic Endometriosis Pain and Bleeding Diary (EPBD)	Deal et al. (11)	Baseline and longitudinal pain/bleeding assessment	Construct validity supported; item-total correlations 0.40–0.89; test-retest ICCs 0.59–0.72	Responsiveness and optimal scoring incompletely established
ESM-based ePRO for real-time symptom assessment in endometriosis	Development: van Barneveld et al. (13); psychometric evaluation: van Barneveld et al. (12)	Real-time symptom and within-person trajectory assessment	Strong concurrent validity; good-to-excellent internal consistency; prompt compliance approximately 52%	High-frequency sampling burden and selective adherence

**Table 4:** Broader digital intervention and delivery categories.

Tool category	Data/intervention	Primary clinical use	Supporting evidence	Main limitation
Mobile diaries and self-tracking apps	Daily symptoms, medication, cycle context, flare triggers	Self-management and follow-up preparation	App-quality, self-tracking, and implementation studies support feasibility and longitudinal symptom capture; no single standardized validated instrument represents this category	Sustained engagement, data overload, and variable quality
Digital therapeutics	Structured immersive, behavioural, or educational interventions	Pain and quality-of-life support	Randomized controlled trials provide intervention-level evidence for selected immersive and behavioural digital therapies	Patient selection, durability, and integration with usual care
Remote-care platforms	Symptoms, visit needs, treatment response, patient questions	Tele-follow-up and coordinated review	Evidence is derived mainly from qualitative, feasibility, service-evaluation, and tele-PROM studies rather than instrument-level psychometric validation	Workflow integration, escalation rules, and digital exclusion

The EPBD and ESM-based ePRO are validated measurement instruments. Mobile diaries/self-tracking apps, digital therapeutics, and remote-care platforms represent broader digital intervention or delivery categories and are therefore summarized using clinical, feasibility, or implementation evidence rather than instrument-level psychometric reliability and validity. ePRO, electronic patient-reported outcome.

## 7 Mhealth, telemedicine, digital decision support, and self-management

### 7.1 Mobile applications and targeted digital interventions

mHealth applications can provide education, symptom tracking, medication reminders, care navigation, and targeted interventions. Systematic reviews and app searches show substantial variation in quality, evidence, usability, and privacy practices (19, 28, 37). Problem-specific interventions may be more clinically interpretable than feature-rich general apps. For example, the Odeya intervention targets sexual distress and has been evaluated for adherence, acceptability, and outcomes (51). Self-tracking research also shows that apps reshape expectations and relationships with care, not merely record symptoms (52). Quality assessments of endometriosis apps reinforce the need for transparent content, clinical review, and evidence-based design (53).

### 7.2 Telemedicine and remote follow-up

Telemedicine may improve continuity for counselling, treatment review, multidisciplinary support, and selected preoperative or postoperative tasks. Evidence from endometriosis care, infertility services, adolescent gynecology, virtual preoperative evaluation, and telephone consultations suggests that remote care can be acceptable or feasible in defined contexts (20, 54–57). Generalizability is limited because these studies address different populations and workflows. Each remote pathway should specify which tasks can be managed virtually, which require physical examination or imaging, and how deterioration or uncertainty triggers in-person care (26–28).

### 7.3 Health literacy, online information, and decision support

Digital tools may strengthen health literacy, self-care, and preparation for shared decisions (38). However, access to information does not guarantee accuracy. Cross-platform video analyses and evaluations of generative AI responses show that quality and reliability can vary (32, 33). Patient-facing content should therefore state its evidence base, intended use, uncertainty, and escalation advice. Digital patient decision aids are an emerging field, but current evidence includes protocol-stage work, so claims of effectiveness should remain cautious (58).

The most defensible role for mHealth and telemedicine is not autonomous care. It is structured support that improves information continuity, prepares shared decisions, reduces avoidable access barriers, and maintains contact between visits while preserving clinical accountability (19, 20, 26, 28, 37–39).

## 8 Fertility management, treatment follow-up, and recurrence reassessment

### 8.1 Goal-aligned longitudinal information

Endometriosis management often requires decisions that change with reproductive intentions, age, prior surgery, ovarian reserve, symptom burden, treatment acceptability, and access to care. Longitudinal management may include surgery, hormonal suppression, analgesic or supportive treatment, and emerging non-hormonal approaches; none should be assumed to eliminate the need for subsequent symptom and fertility reassessment (1). Surgical history should be captured with procedure type, lesion phenotype/site when available, postoperative response, complications, and later concerns such as recurrence or adhesion-related pain. Hormonal treatment should be linked to exposure, adherence, symptom response, adverse effects, and reasons for discontinuation, including clinically relevant effects on mood or bone health when applicable. Patient-initiated lifestyle, dietary, or nutritional strategies may also be recorded because they can influence symptom experience and treatment preferences, although evidence for specific interventions remains heterogeneous and should not be interpreted as established curative therapy (59, 60). Digital systems may help maintain a shared longitudinal profile and make changes in goals visible. The system should organize information for counselling rather than convert complex reproductive decisions into a single prediction score (1, 28, 61, 62).

### 8.2 Fertility-related prediction and counselling

Guideline-based assessment remains the foundation for fertility counselling (1). Data-driven models have been explored for natural conception and live-birth outcomes after assisted reproduction (61, 62). These studies demonstrate potential for individualized risk communication, but models must be evaluated for calibration, transportability, decision impact, and the way uncertainty is communicated. A prediction should inform discussion; it should not determine treatment without consideration of partner factors, tubal factors, disease phenotype, previous treatment, and patient preferences (21, 22, 24).

### 8.3 Treatment follow-up and recurrence reassessment

Longitudinal follow-up should extend beyond lesion recurrence or imaging outcomes. A multidimensional reassessment may combine pain and other symptoms, functional impact, medication and hormonal exposure, treatment response and adverse effects, surgical history, relevant coexisting conditions, reproductive goals, and selected lifestyle context, with imaging or laboratory evaluation used when clinically indicated. Where inflammatory biomarkers, wearable-derived signals, or other passive measures are considered, their analytical validity, incremental clinical value, and actionable thresholds should be established before they are incorporated into routine monitoring. Machine-learning approaches have been explored for long-term recurrence after laparoscopic treatment combined with GnRHa (63). This is a high-risk application because recurrence definitions, prediction horizons, missingness, and response rules can substantially change the meaning of an alert. A digital alert should prompt review and possible investigation; it should not establish recurrence (21, 22, 24).

The near-term opportunity is therefore a data-linked follow-up pathway that makes treatment history, reproductive goals, symptom and functional trajectories, relevant comorbidities, patient-initiated self-management strategies, and reassessment events visible. Predictive models may be added only when their incremental value and consequences have been prospectively evaluated (23, 24). Figure 5 integrates fertility goals, treatment history, symptom trajectories, and recurrence reassessment within a longitudinal pathway, while Table 5 summarizes the corresponding clinical scenarios, data requirements, and limitations.

**Figure 5:**
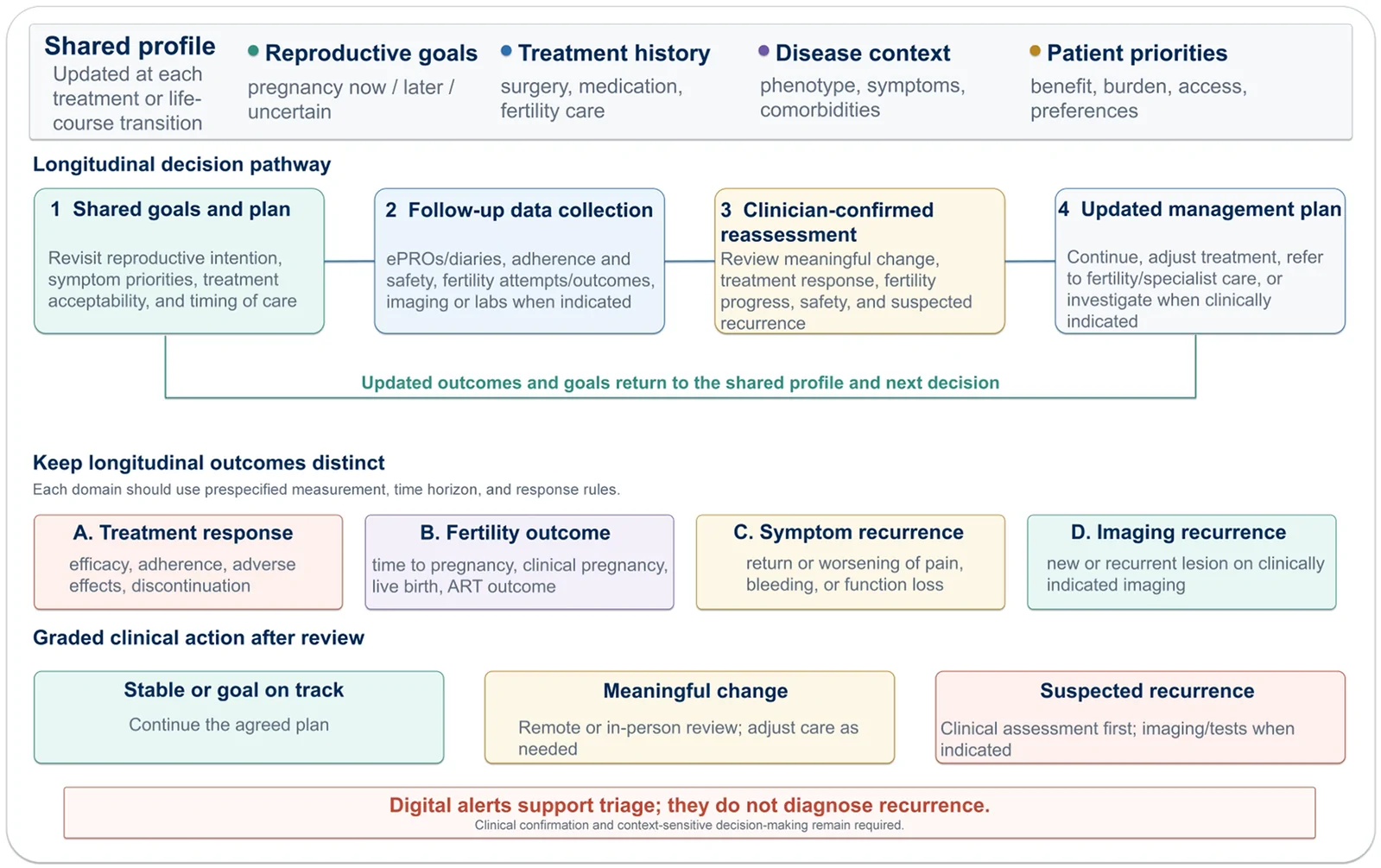
Goal-aligned longitudinal pathway for fertility management, treatment follow-up, and recurrence reassessment in endometriosis. A shared longitudinal profile integrates reproductive goals, treatment history, disease context, and patient priorities and is updated across treatment and life-course transitions. Follow-up data support clinician-confirmed reassessment and revision of the management plan. Treatment response, fertility outcomes, symptom recurrence, and imaging recurrence are retained as distinct longitudinal outcome domains with prespecified measurement and response criteria. Digital alerts may support triage and prompt clinical review but do not establish recurrence, which requires clinical assessment and, when indicated, imaging or other investigations.

**Table 5:** Digital support for fertility management, follow-up, and recurrence reassessment.

Scenario	Core longitudinal data	Decision or prediction target	Potential value	Principal limitation	Representative evidence
Fertility counselling	Reproductive intentions, age, clinical context, prior treatment, fertility assessment	Natural conception or ART-related outcomes	Structured and individualized counselling	Outcome heterogeneity, calibration, and risk of over-reliance on model output	(1, 21, 22, 61, 62)
Post-treatment follow-up	Symptoms, medication, procedures, treatment response, imaging when indicated	Response, adverse effects, and need for review	Continuity and timely reassessment	Follow-up loss, burden, and unclear response thresholds	(1, 20, 26, 27, 63)
Recurrence reassessment	Longitudinal clinical and patient-reported data	Risk update and prompt for clinical review	Earlier recognition of clinically meaningful change	Variable recurrence definitions and sparse prospective validation	(1, 21, 22, 24, 63)

ART, assisted reproductive technology.

## 9 EHR, real-world data, and longitudinal integration

### 9.1 Phenotype validation and data quality

EHRs, administrative data, registries, and RWD can support cohort identification, pathway analysis, long-term outcome tracking, and model evaluation (31). They are not automatically valid. EHR and administrative-data validation studies show that case definitions and algorithms must be tested before they are used for research, prediction, or service evaluation (9, 10). Changes in diagnostic guidance can also alter coding and observed incidence, complicating comparisons across time and settings (14, 31, 64).

### 9.2 Integration with patient-generated and wearable-derived data

The future architecture will likely link validated EHR phenotypes with ePROs, imaging, treatment history, and selected patient-generated data (14, 31). Wearable or passive measures may add cycle context, activity, sleep, or biomarker-related information, but their role in endometriosis is less developed. A wearable in-pad diagnostic demonstrates technological feasibility, while large digital cohorts show the scale at which menstrual information may be collected (44, 65). Neither example establishes routine clinical utility. Data provenance, missingness, consent, and the meaning of passive signals must remain explicit (14, 28, 29).

### 9.3 From data integration to a learning health system

A learning health system requires more than technical linkage. Data must be summarized into clinically interpretable information, returned to care teams within existing workflows, and used to evaluate outcomes and inequities. Interoperability should reduce duplicate entry and fragmentation rather than create another parallel platform. Governance should define who can access sensitive reproductive-health data, how long data are retained, how model updates are controlled, and how patients can understand or contest automated inferences (14, 28, 29, 31).

### 9.4 Target state: clinical coherence and accountable care

The target state of a digitally enabled endometriosis pathway should not be maximal data capture, but clinical coherence: a longitudinally consistent and clinically meaningful representation of the patient that integrates biological and clinical information, patient-expressed experience, and interpretive clinical judgment (34). In this framework, digital tools have value when they preserve clinically relevant knowledge across primary care, specialist assessment, imaging, treatment transitions, fertility planning, and long-term follow-up. Fragmented data streams that are not interpreted, assigned to a responsible reviewer, or connected to an action pathway may increase information volume without improving care. Clinical coherence therefore provides a system-level criterion for judging whether digital health reduces fragmentation or merely digitizes it (34).

## 10 Implementation, ethics, equity, and regulation

### 10.1 Implementation is part of the evidence

Digital tools often fail because they are not integrated into clinical workflow, reimbursement, staffing, governance, or patient routines. Implementation evidence is therefore part of the scientific evidence base. Studies should report where the tool sits in the pathway, who reviews its output, how long review takes, what action thresholds are used, how exceptions are handled, and whether the intervention changes clinical decisions or outcomes (23, 24, 26–28, 31).

### 10.2 Privacy, consent, security, and data minimization

Endometriosis digital health may involve pain, menstrual cycles, fertility intentions, sexual function, mental health, medication, imaging, surgery, location, and patient-generated records. Consent should be specific enough for patients to understand what is collected, how it is used, who can access it, whether secondary use is permitted, and what action may follow. Data minimization, role-based access, secure transfer, auditability, and clear retention policies should be treated as design requirements rather than late-stage compliance tasks (24, 28, 29).

### 10.3 Equity, accessibility, and digital burden

Digital access is shaped by smartphone availability, internet connectivity, language, disability, digital literacy, socioeconomic circumstances, geography, and health-system navigation. A tool that performs well in a highly resourced population may widen diagnostic or treatment inequalities when deployed more broadly. Burden is also an equity issue: frequent data entry, complex interfaces, repeated alerts, or unclear feedback may exclude patients with severe symptoms, limited time, or lower digital confidence. Co-design, multilingual interfaces, accessibility testing, and non-digital alternatives should be built into evaluation (24, 28).

### 10.4 Regulation, accountability, and human oversight

Oversight should match intended use and risk (24, 29, 30). Educational content, symptom diaries, decision aids, triage models, diagnostic AI, and recurrence alerts require different evidence and regulatory controls. Broader reviews of AI in women's health emphasize the need for validation, governance, and responsible adoption (66). Digital decision-aid research in endometriosis is emerging, but protocol-stage work should not be interpreted as evidence of clinical effectiveness (58). For higher-risk tools, responsibility for review, override, incident reporting, model updates, and post-deployment monitoring must be explicit. At the system level, these responsibilities can be conceptualized through Duty of Care Governance, in which the organization remains accountable for sensing clinically relevant signals, assigning review responsibility, defining escalation pathways, closing the feedback loop, and preserving continuity when care is distributed across clinicians, services, and digital technologies (67). Table 6 consolidates the principal translation barriers and the priority actions required to move digital tools from feasibility studies toward responsible clinical use (23, 24, 29, 30).

**Table 6:** Translation barriers and priority research directions.

Barrier	Why it matters	Priority action	Relevant domain	Representative evidence
External validation and clinical utility	Many models remain internally validated or are evaluated only by discrimination	Independent validation, calibration, decision analysis, and prospective workflow evaluation	AI/ML diagnosis and prediction	(18, 21–24, 35, 36, 45)
Adherence and monitoring burden	Longitudinal value depends on sustained, representative use	Co-design, low-burden schedules, missing-data analysis, and clinically meaningful feedback	ePROs, mHealth, and tele-follow-up	(11–13, 19, 25–27, 37, 51, 52)
Privacy, safety, and equity	Sensitive reproductive-health data and unequal digital access can create harm	Data minimization, privacy-preserving design, accessibility testing, subgroup audits, and non-digital alternatives	Patient-facing tools and AI-enabled pathways	(19, 24, 28, 29, 37, 66)
Interoperability and accountability	Fragmented apps and unclear responsibility limit clinical uptake	Validated phenotypes, shared standards, audit trails, defined escalation, and named clinical ownership	EHR/RWD and implementation	(9, 10, 14, 24, 28, 30, 31, 64)

AI, artificial intelligence; EHR, electronic health record; ePRO, electronic patient-reported outcome; ML, machine learning; RWD, real-world data.

## 11 Discussion

### 11.1 Principal findings and contribution

This Review integrates a technically diverse and clinically fragmented literature through a life-course framework. Its central contribution is to connect early recognition, assisted diagnosis, symptom monitoring, intervention delivery, treatment and fertility follow-up, recurrence reassessment, and self-management within one longitudinal pathway. The analysis indicates that digital health is most credible when it improves information continuity and supports defined clinical actions. Isolated model performance, extensive app functionality, or large volumes of patient-generated data do not by themselves establish clinical value.

The evidence is uneven in both volume and maturity. AI/ML research is prominent but remains dominated by model development and internal validation (18, 21–24, 35, 36). ePROs and electronic diaries are closer to clinical practice because they address information that already matters to care, although response thresholds and workflow integration remain variable (11–13, 20, 25–27). Digital pain interventions and selected telehealth programmes have randomized or prospective evidence, but the range of evaluated interventions is still limited (16, 17, 39). EHR/RWD, interoperability, and implementation governance are essential to scale, yet they are less developed than individual tools (9, 10, 14, 28, 31).

A further strength of this clinical-function approach is that it permits patient-generated data, AI/ML, mHealth, telemedicine, digital therapeutics, and EHR/RWD to be appraised as complementary components of a care pathway rather than as isolated technology categories.

### 11.2 Clinical implications and priority use cases

Near-term use cases are those that strengthen existing clinical processes without claiming autonomous diagnosis. These include structured symptom capture before consultation, brief ePRO monitoring linked to treatment review, remote support with clear escalation rules, patient education with transparent evidence, and shared longitudinal records that make treatment history and reproductive goals visible. Such uses may improve preparation, continuity, and timing even when they do not directly change diagnostic accuracy (11–13, 19, 20, 25–28, 37–39).

Intermediate-term applications include AI-assisted imaging, multimodal risk triage, fertility-related prediction, and recurrence alerts. These may provide incremental value, but only if independent validation is followed by prospective assessment of decisions, outcomes, workload, and equity (21–24). Standalone population screening, fully automated diagnosis, and unsupervised recurrence determination are not supported by the current evidence (1, 8, 24, 36, 61–63).

### 11.3 Trade-offs and unintended consequences

Every potential benefit has a corresponding risk. Earlier triage may shorten delay but can also increase false reassurance or over-referral. Continuous monitoring may support personalized care but can generate burden, alert fatigue, and selective missingness. Remote access may reduce travel while excluding people with limited connectivity or digital confidence. High-performing models may fail under spectrum shift, domain drift, or changes in imaging practice. Personalization may improve relevance while increasing privacy risk or obscuring accountability. These trade-offs should be assessed explicitly rather than treated as secondary implementation issues (24, 26–29).

### 11.4 Limitations of this review

A narrative approach was selected because the review question spans heterogeneous technologies, clinical functions, data types, study designs, and stages of translation that are not readily addressed by a single pooled-effect framework. This design allows the literature to be interpreted through a common clinical pathway and implementation lens, but it also limits the certainty and reproducibility of the conclusions. The search and selection process was structured but purposive rather than exhaustive; duplicate independent screening, formal risk-of-bias assessment, publication-bias assessment, and quantitative pooling were not performed. Consequently, relevant studies may have been missed, and the relative prominence of specific technologies in this Review should not be interpreted as a quantitative estimate of the underlying evidence base.

Evidence directness also varies. Some studies are endometriosis-specific, whereas others inform measurement, interoperability, governance, or implementation from broader digital-health settings. The included literature is heterogeneous in population, reference standards, outcomes, follow-up duration, and validation quality, which limits direct comparison across modalities. In addition, digital technologies, datasets, regulatory expectations, and commercial products change rapidly; therefore, conclusions regarding readiness may require updating as new external validations and prospective implementation studies emerge. These limitations particularly constrain claims about comparative effectiveness, generalizability, equity, and routine clinical impact.

### 11.5 Future research priorities

Future studies should move from isolated feasibility toward pathway-level evaluation. AI/ML research should prespecify intended use, report calibration and subgroup performance, perform independent external validation, and assess clinical utility prospectively. ePRO research should define meaningful change, alert thresholds, response ownership, burden, and missingness. mHealth and digital therapeutics should be evaluated for durable clinical outcomes, accessibility, privacy, and integration with usual care rather than engagement alone. EHR/RWD studies should use validated phenotypes, transparent data models, and explicit governance (14, 21–28, 30, 31).

The next generation of digital endometriosis care should link patient-generated data, clinical records, imaging, treatment history, fertility goals, and long-term outcomes into an interoperable record that supports shared decisions and timely reassessment. Human oversight, patient involvement, equity assessment, and accountability should be treated as core design requirements. The goal is not fully automated care, but better-timed, better-informed, and more patient-centred care (14, 24, 28, 29).

## 12 Conclusion

Digital health may address persistent challenges in endometriosis, including delayed recognition, fragmented diagnostic pathways, recurrent symptoms, insufficient follow-up, fertility-related decisions, and limited self-management support. Its greatest potential lies in a clinician-supervised life-course infrastructure rather than a collection of isolated tools. AI/ML, mHealth, ePROs, telemedicine, digital therapeutics, EHR/RWD, and patient education each provide partial value. Progress will depend on connecting them through validated, interoperable, equitable, and accountable pathways and on moving from feasibility and internal model performance toward external validation, prospective evaluation, and implementation-ready care.
