# Supplementary material for: Digital health-enabled life-course management of endometriosis: from early recognition and assisted diagnosis to symptom monitoring and self-management
Source: Front Med (Lausanne). 2026 Oct 6;13:1951037. doi: 10.3389/fmed.2026.1951037 (PMC13693527; doi:10.3389/fmed.2026.1951037)
Supplement: Supplementary file 1 [file Table1.docx]

**Supplementary Table S1.**

**4.1 PubMed/MEDLINE**

Core digital-health block: ("endometriosis"[Title/Abstract] OR "Endometriosis"[Mesh]) AND ("digital health"[Title/Abstract] OR "artificial intelligence"[Title/Abstract] OR "machine learning"[Title/Abstract] OR "mobile health"[Title/Abstract] OR mHealth[Title/Abstract] OR "electronic patient-reported outcome*"[Title/Abstract] OR ePRO[Title/Abstract] OR telemedicine[Title/Abstract] OR telehealth[Title/Abstract] OR "digital therapeutic*"[Title/Abstract] OR "electronic health record*"[Title/Abstract] OR "real-world data"[Title/Abstract] OR wearable*[Title/Abstract] OR "digital decision support"[Title/Abstract])

Early-recognition / diagnostic-pathway supplementary block: ("endometriosis"[Title/Abstract] OR "Endometriosis"[Mesh]) AND ("early detection"[Title/Abstract] OR screening[Title/Abstract] OR "diagnostic delay"[Title/Abstract] OR "diagnostic imaging"[Title/Abstract] OR ultrasonograph*[Title/Abstract] OR ultrasound[Title/Abstract] OR MRI[Title/Abstract] OR "magnetic resonance imaging"[Title/Abstract] OR "symptom checker"[Title/Abstract] OR triage[Title/Abstract]) AND (digital[Title/Abstract] OR algorithm*[Title/Abstract] OR "artificial intelligence"[Title/Abstract] OR "machine learning"[Title/Abstract] OR app[Title/Abstract] OR electronic[Title/Abstract])

**4.2 Web of Science Core Collection**

TS=(endometriosis) AND TS=("digital health" OR "artificial intelligence" OR "machine learning" OR "mobile health" OR mHealth OR "electronic patient-reported outcome*" OR ePRO OR telemedicine OR telehealth OR "digital therapeutic*" OR "electronic health record*" OR "real-world data" OR wearable* OR "digital decision support")

Supplementary diagnostic block: TS=(endometriosis) AND TS=("early detection" OR screening OR "diagnostic delay" OR "diagnostic imaging" OR ultrasonograph* OR ultrasound OR MRI OR "magnetic resonance imaging" OR "symptom checker" OR triage) AND TS=(digital OR algorithm* OR "artificial intelligence" OR "machine learning" OR app OR electronic)

**4.3 Scopus**

TITLE-ABS-KEY(endometriosis) AND TITLE-ABS-KEY("digital health" OR "artificial intelligence" OR "machine learning" OR "mobile health" OR mHealth OR "electronic patient-reported outcome*" OR ePRO OR telemedicine OR telehealth OR "digital therapeutic*" OR "electronic health record*" OR "real-world data" OR wearable* OR "digital decision support")

Supplementary diagnostic block: TITLE-ABS-KEY(endometriosis) AND TITLE-ABS-KEY("early detection" OR screening OR "diagnostic delay" OR "diagnostic imaging" OR ultrasonograph* OR ultrasound OR MRI OR "magnetic resonance imaging" OR "symptom checker" OR triage) AND TITLE-ABS-KEY(digital OR algorithm* OR "artificial intelligence" OR "machine learning" OR app OR electronic)

**4.4 Google Scholar**

• endometriosis "digital health"

• endometriosis "artificial intelligence" diagnosis

• endometriosis "early detection" digital

• endometriosis "diagnostic delay" app OR digital

• endometriosis telemedicine ePRO

• endometriosis wearable
